# Detecting horizontal gene transfer: a probabilistic approach

**DOI:** 10.1186/s12864-019-6395-5

**Published:** 2020-03-05

**Authors:** Gur Sevillya, Orit Adato, Sagi Snir

**Affiliations:** Dept. of Evolutionary and Environmental Biology, University of Haifa, Haifa, 3498838 Israel

**Keywords:** Gene order, Horizontal gene transfer, Phylogenetics

## Abstract

**Background:**

Horizontal gene transfer (HGT) is the event of a DNA sequence being transferred between species not by inheritance. HGT is a crucial factor in prokaryotic evolution and is a significant source for genomic novelty resulting in antibiotic resistance or the outbreak of virulent strains. Detection of HGT and the mechanisms responsible and enabling it, is hence of prime importance.Existing algorithms rely on a strong phylogenetic signal distinguishing the transferred sequence from its recipient genome. Closely related species pose an even greater challenge as most genes are very similar and therefore, the phylogenetic signal is weak anyhow. Notwithstanding, the importance of detecting HGT between such organisms is extremely high for the role of HGT in the emergence of new highly virulent strains.

**Results:**

In a recent work we devised a novel technique that relies on loss of synteny around a gene as a witness for HGT. We used a novel heuristic for synteny measurement, SI (Syntent Index), and the technique was tested on both simulated and real data and was found to provide a greater sensitivity than other HGT techniques. This synteny–based approach suffers low specificity, in particular more closely related species. Here we devise an adaptive approach to cope with this by varying the criteria according to species distance. The new approach is doubly adaptive as it also considers the lengths of the genes being transferred. In particular, we use *Chernoff* bound to decree HGT both in simulations and real bacterial genomes taken from EggNog database.

**Conclusions:**

Here we show empirically that this approach is more conservative than the previous *χ*^2^ based approach and provides a lower false positive rate, especially for closely related species and under wide range of genome parameters.

## Background

**Fig. 1:**
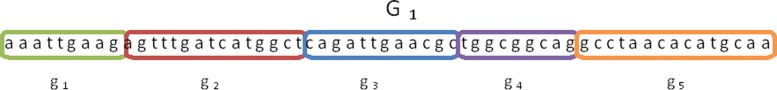
A genome is viewed as a sequence of genes while a gene is a sequence of nucleotides

Genomes of bacteria and archaea are characterized by extensive gene mobility between species that is crucial not only for evolution of genome architecture but also for the functionality of prokaryotic organisms [12]. The principal mechanism accounting for gene mobility is horizontal gene transfer (HGT) [6, 14, 18] in which a gene (or a group of genes) of a donor species being acquired by a recipient organism. HGT, to a large extent, is mediated by viruses (bacteriophages), plasmids, transposons and other mobile elements. The genetic interpretation of this event is a gene being copied from the donor genome to the recipient genome (see Fig. 1).

The study of the HGT is of paramount importance for several reasons. First, from medical perspective, HGT plays a major role in the emergence of new human diseases, as well as promoting the spread of antibiotic resistance in bacteria species [21]. From the fundamental, evolutionary standpoint, HGT links distant branches in the tree of life, turning it into an evolutionary network [6, 30]. Genetically, HGT is an important, if not the primary source of new genes that are acquired by bacteria and archaea and often result in adaptations to new environments and conditions [5]. Recent advances of comparative genomics and especially metagenomics indicate that the complexity of the genetic material that is horizontally transferred is vast and exceeds by several orders of magnitude the complexity of the set of conserved genes that are mostly vertically inherited [7]. Therefore, identification of HGT can shed light on many significant evolutionary processes that cannot be explained by the traditional tree–like approach.

Currently, there are two prevailing methods for detecting HGT. The *phylogeny based approach* takes a relatively large set of homologous (originated from a common ancestor) coding sequences, constructs their corresponding phylogeny, and contrasts it to the phylogeny of their originating species. When conflicts are found between the two trees, they are reconciled by introducing HGTs (see e.g. [13, 17]). While this approach has the advantage of identifying relatively old events, the approach is based on a very stringent assumption of where to seek the events. Finally, it also requires a *multiple* alignment of the sequences and inferring a reliable species tree (two major problems by themselves [31]). The *composition based approach* contrasts genomic sequences of different compositional structure such as G+C content, dinucleotide frequencies or codon usage biases, striving to infer different origins (e.g. [3, 11, 18, 20]). This approach suffers from the fact that the species involved might share similar compositional patterns. Moreover, the length of a transferred segment may be too short to reliably reveal these differences. As concluded in [16], “atypical G+C content and pattern of codon usage are not reliable indicators of horizontal gene transfer events”.

Both the phylogenetic and the sequence based approaches rely on a strong enough signal for the HGT. Such a signal may not exists when dealing with closely related species or even strains of the same species. In two recent works [1, 24], we have defined the notion of *synteny index* (SI) between two genomes (species) and used it as a marker of evolutionary footprints. Gene synteny [8, 23] is the conservation of gene order across species along the evolutionary course. Synteny (or lack of) was already employed for defining a distance measure between genomes (species), counting the minimal number of operations to transform one genome to another [4]. Nevertheless this distance is irrelevant in the context of a particular single gene. In contrast, SI measures how much a gene, orthologous to the two species, is in its “natural place”, or in other words, shares the same neighborhood in both genomes. In [24] we averaged the SI over the whole genome and used it to infer evolutionary distance. We next aimed at identifying HGTs between closely related species by means of SI [1]. The technique relies on the *constant relative mutability* (CRM) property that asserts that the ratio between the mutational rates of genes is maintained across species (even if the rates themselves change along time/species). The method was compared to several representative HGT methods, both phylogenetic methods such as RIATA–HGT and *PhylTR* [19, 27], and sequence based– *HGT–DB* [11]. It was also employed to real biological data, the three strains of *E. Coli* that were studied in [28] and were found to exhibit a very high rate of HGT. Understanding and detecting HGT within the strains, could be of great importance, for instance in understanding the origin of pathogenicity of certain pathogenic strains, particularly those whose ancestors were not pathogenic.

In this work we make another step forward by formulating the problem in a statistical framework. This allows us to apply probabilistic tools that are more advanced than those employed in [1]. We start by providing a tool to measure the significance of SI of a given gene over the background noise of its hosting genomes. Next we apply bounds on large deviations to assess the probability of a gene being transfered to the hosting genome or it has existed there since divergence from the donor species. This check requires the transformation between two spaces: one, in which the CRM exists and allows us to derive expected values for the gene distance, and another, in which we can assess the likelihood of the observed distance with respect to the expected one. We conducted a simulation study where we compared the current approach with that of [1] and showed it provides a greater specificity (i.e., lower false positive rate). All steps of the proposed method are very efficient as they operate between pairs of orthologous genes and therefore the complexity of the method is at most quadratic in the size of the taxa set.

## Definitions and methods

**Fig. 2:**
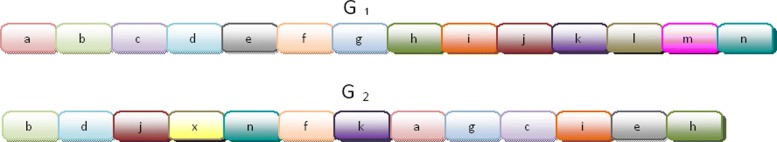
Comparing *G*_1_ with *G*_2_ for *k*=3: *SI*(*g*,*G*_1_,*G*_2_)=3, *SI*(*x*,*G*_1_,*G*_2_)=0, *SI*(*ℓ*,*G*_1_,*G*_2_)=0

We now define our working model that will serve to locate HGT between genes. A genome *G* is a sequence (although we sometimes treat it as an ordered set) of genes (*g*_1_,*g*_2_,…,*g*_*n*_) and each gene is a sequence of DNA letters. That is, our view of a genome is at a resolution of genes, and of a gene at a resolution of nucleotides (See Fig. 2). The *k–neighborhood* of a gene *g*_0_ in genome *G*, *N*_*k*_(*G*,*g*_0_) is the set of genes at distance at most *k* from *g*_0_ in *G* (i.e. at most *k* genes upstream or downstream, not including gene *g*_0_ itself). The *core set* (i.e., intersection) of genomes *G*_1_ and *G*_2_ is *G*_1_∩*G*_2_, and the symmetric difference of *G*_1_ and *G*_2_ is *G*_1_△*G*_2_=*G*_1_∪*G*_2_∖*G*_1_∩*G*_2_.

The conservation of the order between genes in $\cap _{G_{1},G_{2}}$ is called *synteny*. Let $g_{0} \in \cap _{G_{1},G_{2}}$. Then the *ksynteny index* (*k*-SI), or just SI when it is clear from the context, of *g*_0_ in *G*_*i*_,*G*_*j*_ is the number of common genes in the *k* neighborhoods of *g*_0_ in both *G*_*i*_ and *G*_*j*_: *SI*(*g*_0_,*G*_*i*_,*G*_*j*_)=|*N*_*k*_(*G*_*i*_,*g*_0_)∩*N*_*k*_(*G*_*j*_,*g*_0_)|. For the sake of completeness, for *g*_0_∉*G*_*i*_∩*G*_*j*_, *SI*(*g*_0_,*G*_*i*_,*G*_*j*_)=0. See Fig. 2 for illustration.

A genome undergoes events of gene gain and loss in which genes are added or removed respectively. As we are focused in the core set of genes that are common to two organisms, we are not interested in the latter processes. Every gene undergoes a process of sequence evolution according to some stochastic evolutionary model [9]. The evolutionary model we consider is such that the nucleotides along a gene are identically and independently distributed (IID). The value of the nucleotide is the *state* (we sometimes use just “nucleotide” to denote its state). A *single mutation* (or *point mutation* or just a mutation for short) is the event of a nucleotide changing its value to a different one (for reasons of simplicity, we use the term ’mutation’ as for a point mutation that occurs and then gets fixed, i.e., ’a nucleotide substitution’). An *evolutionary model*$\mathcal {M}$ models the (stochastic) process of mutations occurring at a site as a function of *mutation rates*$\mathcal {a}_{i,j}$ modeling the rate of transitions from state *i* to *j*, and a specified time period *t*. We use the *transition* notation in the context of Markov chains and note that it has nothing to do with the type of mutation baring the same notation (see [9] for more details). Given $\mathcal {M}$, mutation rates $[\mathcal {a}_{i,j}]$, and a time period *t*, the *transition probability* *p*_*i*,*j*_ from nucleotide *i* to *j* during *t* is uniquely defined by an appropriate function (determined by $\mathcal {M}$). An evolutionary model $\mathcal {M}$ is said to be *time reversible* if it is not possible to determine the direction of time given two states of a nucleotide, separated by a time period *t*. The *evolutionary distance* (or *mutation distance* or simply distance) is the number of mutations separating between two homologous sequences. The *hamming distance* between two homologous sequences counts the number sites with different states. These distances are usually normalized by the length of the sequences and are normally denoted by *d* and *h* respectively. In the Results section, we used the Jukes–Cantor [15] (JC) evolutionary model. See more specific details in “Results” section.

A *horizontal gene transfer* (HGT) is the event in which a gene of a genome, the *donor genome*, being copied and inserted at some position at another genome, the *recipient genome*. Since we view the genome as a circled sequence of genes, the new gene is always between two genes.

## Results

Consider two genomes after speciation event. Gene order, synteny, in the two genomes is nearly the same, and hence orthologous genes have almost the same neighboring genes in the two genomes. Due to events such as HGT, this similarity decreases as the time since the divergence event grows. Hence, between closely related species (and in particular strains of a species), if a gene has exceptionally low SI, we might suspect it has undergone HGT. We denote these genes as *SI HGT suspected*.

### Significant SI HGT suspected genes

We want to verify that SI suspected genes are indeed a result of a HGT and not a background noise. When the core set of genes is small, with some probability, low SI is observed even if a gene is in its original location. This is due to gene loss events around that gene. If all genes around gene *g* were lost, *g* has SI zero without being transferred. We will associate a confidence value with every SI, and set a threshold value *δ*_*SI*_ for obtaining low SI by random (i.e. not by HGT rather simply by gene gain/loss).

#### **Lemma 1**

Consider two genomes *G*_1_ and *G*_2_. Let *g* be a gene in the core set of *G*_1_ and *G*_2_ ($g \in \cap _{G_{1},G_{2}}$) with |*G*_1_|=|*G*_2_|=*n* and let *δ*_*SI*_ be an arbitrary probability. Then with probability at most *δ*_*SI*_ we expect to find by chance SI of size:

1 $$ SI < \frac kn \left (2n-|G_{1} \bigtriangleup G_{2}|\right) -\sqrt{ -k \log_{e} \delta_{SI}}  $$

#### *Proof*

We denote a gene *g*_*i*_ as *singular* if *g*_*i*_∈*G*_1_△*G*_2_.

**Observation** The probability of hitting a singular gene by chance is $ \frac {|G_{1} \bigtriangleup G_{2}|}{2n}$. □

*Proof* Since by assumption the length of both genomes is *n*, the symmetric difference *G*_1_△*G*_2_ is partitioned equally on both genomes (since the core set $\cap _{G_{1},G_{2}}$ exists on both). Since a randomly chosen gene from a given genome is either from $\cap _{G_{1},G_{2}}$ or *G*_1_△*G*_2_ and $n=|\cap _{G_{1},G_{2}}| +\frac {|G_{1} \bigtriangleup G_{2}|}{2}$, the result follows. □

Henceforth we will denote by *p* this probability, i.e. $p = \frac {|G_{1} \bigtriangleup G_{2}|}{2n}$, and note that *p* is easily calculated.

We now focus on genes *g*_*i*_ for 1≤*i*≤2*k* in the *k*–neighborhood of gene *g*. Let *X*_*i*_=1−*p* if gene *g*_*i*_ is singular, and −*p* otherwise, and let $X=\sum X_{i}$. Then we observe that Pr[*X*_*i*_=1−*p*]=*p* and Pr[*X*_*i*_=−*p*]=1−*p*. Hence, *E*[*X*_*i*_]=0 and *X* follows a distribution *B*(2*k*,*p*)−2*kp* where *B*(*n*,*p*) is the usual binomial distribution (this is a good approximation given the reasonable assumption that *k*≪*n*).

Our goal is to bound the probability of deviation from the expected value and seeing a low SI only by random. The distribution of *X* allows us to apply Chernoff bound [2](Thm A.4) asserting

2 $$ \Pr[ X > a ]\leq e^{-2a^{2}/n}.  $$

We are seeking for the minimal *a* such that this probability is smaller than *δ*_*SI*_. In our case *n* is 2*k* and hence we set

3 $$ e^{-2a^{2}/2k} = \delta_{SI} \Rightarrow a = \sqrt{-{k}\log_{e} \delta_{SI}},  $$

yielding:

4 $$ \Pr\left (X > \sqrt{- k \log_{e} \delta_{SI}}\right)\leq \delta_{SI}.  $$

Note that *X* counts the observed number of genes in only a single *k*–neighborhood of *g* (they are *not* necessarily singular) minus their expected number– 2*kp*. That is *X*=2*k*−*SI*−2*kp*, where 2*k*−−*SI* is the number of genes in only a single *k*–neighborhood of *g*.

If we substitute in (4) *X*=2*k*−*SI*−2*kp* we obtain:

5 $$\begin{array}{*{20}l} & \Pr\left (2k - SI-2kp > \sqrt{- k \log_{e} \delta_{SI} }\right)\\ =& \Pr\left (SI < 2k(1-p)- \sqrt{-k \log_{e} \delta_{SI}} \right)< \delta_{SI}. \end{array} $$

Back substituting $p =\frac {|G_{1} \bigtriangleup G_{2}|}{2n}$, and the result follows.

Lemma 1 allows us to infer about the increase in the strength of the evidence. For that we equate *a* in Eq. (2) to *X*. As *n* in Eq. (2) is set constant, the only variable component is the SI in *X* (and in *a*) yielding the following:

#### **Corollary 1**

The significance of the evidence grows exponentially in the SI.

Lemma 1, provides an upper bound on the SI scores we expect to see by chance. However, we should be careful here. For some combinations of *p* and *δ*_*SI*_ our neighborhood may not be large enough. For instance, for |*G*_1_△*G*_2_|=1600, *n*=1000, and *δ*_*SI*_=0.05, a neighborhood of 10 is not enough, since by our bound, under that probability we expect to see *SI*<−1.47 by chance, but *k*=30 does suffice (*SI*<2.5). If we increase |*G*_1_△*G*_2_| to 1800 (i.e. *p*=0.9), then even *k*=60 is not enough. We therefore conclude:

#### **Corollary 2**

Let $p=\frac {|G_{1} \bigtriangleup G_{2}|}{2n}$, then for a given *δ*_*SI*_ we must have

6 $$ k \geq -\frac {\log_{e} \delta_{SI} }{4(1-p)^{2}}  $$

#### *Proof*

Since *SI*≥0 must hold, and according to Lemma 1, we get: $0 \ge \frac kn \left (2n-|G_{1} \bigtriangleup G_{2}|\right) -\sqrt { -k \log _{e} \delta _{SI}}$.

Then, $\sqrt { -k \log _{e} \delta _{SI}} \ge \frac kn \left (2n-|G_{1} \bigtriangleup G_{2}|\right)$. We defined before $p = \frac {|G_{1} \bigtriangleup G_{2}|}{2n}$, so we get $\sqrt { -k \log _{e} \delta _{SI}} \ge 2k(1-p)$ and isolation of *k* is trivial. □

### Sifting between other mutational events

In the previous section we derived values under which SI is significant. However, low SI can be a result of other large scale mutational events: A *translocation* is the event where a gene moves to a different location in a genome. A *Duplication* is an identical event only that a copy of the gene remains in the original location.

The following observation follows intuitively from Fig. 2:

#### **Observation 2**

Let *G*_1_ and *G*_2_ be two genomes sharing a common gene *g*. Assume *g* was either translocated or duplicated in *G*_2_ (we assume *g* corresponds to the copied instance rather than the original). Assuming no other large scale mutational events occurred, then *E*[*SI*(*g*,*G*_1_,*G*_2_)]≈ 0.

#### *Proof*

Amuse *G*_1_ and *G*_2_ are two identical genomes, and now gene *g* is translocated in genome *G*_1_. Then, *E*[*SI*(*g*,*G*_1_,*G*_2_)]=0 except if the new position of gene *g* is no further than *k* from its original neighborhood. The probability the new position fulfill this requirement is 4*k*/*n*. For realistic closely related genomes (genome size of 5000 and symmetric difference <0.8, which leads to *k*<10), we get *E*[*SI*(*g*,*G*_1_,*G*_2_)]≈0. □

Indeed, based on SI only, it cannot be distinguished whether gene *d* in Fig. 2 has been horizontally transferred or been translocated. Therefore we cannot rely on low SI as a single evidence for HGT. To distinguish a gene undergone HGT from translocations or duplications, we rely on the fact that a translocated (duplicated) gene has been in its hosting genome since its split from another genome, in contrast to a gene recently acquired through HGT. This implies that the translocated gene was subjected to small scale substitutions (point mutation) for the time period since its split from the other genome. Hence the induced distance between orthologous genes in two genomes, is proportional to the time since their divergence.

#### Constant relative mutability

We now rely on a very basic evolutionary effect recently demonstrated, dubbed as *Universal Pacemaker* (UPM) of genome evolution [25, 26, 29], which serves as a useful approximation for genome evolution processes. The UPM principle states that along every lineage in the evolution of cellular life, most genes change their mutation rate in unison, as if adhering to a universal (but lineage specific) pacemaker. To harness the UPM principle to our purpose, we formulate the problem as follows: We have a gene *g*, *SI suspected* of having undergone HGT between two strains *S*_1_ and *S*_2_, by exhibiting low *SI*(*g*,*S*_1_,*S*_2_)<*δ*_*SI*_ for some threshold value *δ*_*SI*_. We look for a *witness* gene, *w*, and two *reference* organisms *R*_1_ and *R*_2_ under the constraint that *w*∈*R*_1_,*R*_2_,*S*_1_,*S*_2_ and *g*∈*R*_1_,*R*_2_. By the UPM, genes *g* and *w*, although may mutate at different rates, maintain approximately a constant ratio between their rates. More precisely:

##### **Definition 1**

(CRM) Let *g* and *g*^′^ be genes residing in a genome *G* mutating at (not necessarily constant) rates *a* and *a*^′^. Then *g* and *g*^′^ have *constant relative mutability* (CRM) (or alternatively – *conservation*), if at any time, the ratio *ρ*=*a*/*a*^′^ is (approximately) constant.

The result of the CRM phenomenon is that different genes, unless undergone gene specific extraordinary events, maintain the same tree topology and even tree shape. This implies that branch lengths in the corresponding gene trees differ by a multiplicative constant. Note that this property does not contradict rate heterogeneity across genes and also across organisms.

In the following, we operate in two distance spaces: The *Hamming distance* and the *mutation distance* (or *evolutionary distance* or simply, distance). The distances are always defined between two organisms and WRT a certain gene (e.g. *X*_1_,*X*_2_,*g* respectively): *h*_*g*_(*X*_1_,*X*_2_) or *d*_*g*_(*X*_1_,*X*_2_). When it is clear from the context, we ignore either the gene or the two organisms associated with the distance. In the evolutionary distance space, the distance between organisms is the number of mutations separating them, or alternatively, the number of mutations occurred at each lineage since their divergence event.

The Hamming distance is the number of different positions between the genes at the organisms and it is an underestimate for the mutation distance since multiple mutations at a site are unobserved.

To obtain the *expected* mutation distance (we don’t know exactly how many mutations indeed occurred) we use some non–linear *distance correction* function, *d*=*corr*(*h*) and an inverse correction *h*=*corr*^−1^(*d*).

The definition of the CRM phenomenon, operates on the substitution rates of each gene. We however, observe the Hamming distances. To use the CRM phenomenon, we need to convert the Hamming distances to evolutionary distances and then to apply the CRM rule.

##### **Observation 3**

Assume genes *g* and *g*^′^, with mutation rates *r*_*g*_ and $r_{g^{\prime }}$ respectively, satisfy the CRM hypothesis with ratio $\rho =\frac {r_{g}}{r_{g^{\prime }}}$. Let *h*_*g*_ be the Hamming distance WRT gene *g*. Then the expected distance WRT gene *g*^′^, $\delta _{g^{\prime }}$ is

7 $$ \delta_{g^{\prime}} = \frac{ corr (h_{g})}\rho  $$

where *corr* is a distance correction function to correct from the *observed* Hamming distance to the mutation distance.

##### *Proof*

The expected number of substitutions along gene *g* between two sequences *X*_1_,*X*_2_, i.e. the real distance is defined by *d*_*g*_=*corr*(*h*_*g*_(*X*_1_,*X*_2_)). On the other hand, *d*_*g*_=*tr*_*g*_ where *t* is the time separating *X*_1_,*X*_2_, or in other words, twice the time since divergence. Since, by the CRM property ${r_{g'}=\frac {r_{g}}{\rho }}$, we obtain

8 $$ \delta_{g^{\prime}}(X_{1},X_{2}) = tr_{g^{\prime}} = \frac{tr_{g}}\rho = \frac{d_{g}(X_{1},X_{2})}\rho = \frac{ corr (h_{g}(X_{1},X_{2}))}\rho  $$

□

Observation 3 derives the expected distance of a gene *g*^′^ based on the CRM hypothesis and the hamming distance of another gene *g*. If we apply the inverse correction *corr*^−1^ to the expected distance $d_{g^{\prime }}$ we obtain the *expected Hamming distance*$h_{g^{\prime }}$. This is essential since in the hamming distance space we can apply bounds on deviations from the mean that do not apply in the mutation distance space. Therefore, in order to link between the expected and the observed distance WRT gene *g*, we use the following Lemma:

##### **Lemma 2**

Assume genes *g* and *g*^′^, adhering the CRM hypothesis. Let $d_{g^{\prime }}$ be the expected distance WRT to *g*^′^ as derived in Observation *3*. Let $h_{g^{\prime }}$ be the expected Hamming distance obtained by applying the inverse correction on $d_{g^{\prime }}$: $h_{g^{\prime }}=corr^{-1}(d_{g^{\prime }})$. Then, the difference between $h_{g^{\prime }}$ and the observed Hamming distance $\hat h_{g^{\prime }}$ satisfies:

9 $$ \Pr\left[\left| h_{g^{\prime}}- \hat h_{g^{\prime}}\right|>\varepsilon\right]\leq2e^{-2n\varepsilon^{2}},  $$

where *n* is the length a number of nucleotides of *g*^′^.

##### *Proof*

The expected Hamming distance is the probability *p* of observing a difference at a position between the two corresponding sequences. Let $\hat h_{i}$ be an indicator variable indicating a difference at position *i* at both copies of *g*^′^. By definition of $\hat h_{g^{\prime }}$,

$$\hat h_{g^{\prime}} = \frac 1n \sum_{i} \hat h_{i} $$

and

$$E\left[\hat h_{g^{\prime}}\right] = \frac 1n E\left [\sum_{i} \hat h_{i} \right ] = \frac 1n \sum_{i} E\left[ \hat h_{i} \right] = \frac 1n n p = h_{g}, $$

where the second equation is due to linearity of expectation.

Letting $X= \sum _{i} \hat h_{i} $, we can use again Chernoff inequality [2] to bound the deviation of a sum of IID indicator random variables from its mean. For any *ε*>0 holds:

10 $$ \Pr[ |X - E[X] |>\varepsilon n ]\leq 2e^{-2(\varepsilon n)^{2}/n},  $$

and the result follows. □

Observation 3 and Lemma 2 relied on the CRM phenomenon and the constant ratio *ρ* to derive the expected distances and to bound the deviation from them. Since gene *g* is HGT suspected between the two strains, we cannot rely on its distance (Hamming and consequently mutation) to adhere to CRM. Therefore, we look for two *reference organisms* and a *witness* gene *w*, such that both *g* and *w* are present in the strains and the reference organisms. Now we can compute *ρ*=*r*_*g*_/*r*_*w*_ in the reference organisms, and use it to derive the expected distance between the strains WRT *g*. We now state our main theorem for this section that combines all this information with Observation 3 and Lemma 2 to obtain some confidence level on the observed Hamming distance $\hat h_{g}$ between the strains as a function of the distances between the reference organisms.

##### **Theorem 1**

Let *g* be a gene suspected of having undergone HGT between two strain species *S*_1_ and *S*_2_ and let *n*=|*g*| be the length of *g*. Let *w* be a witness gene while *R*_1_ and *R*_2_ are two different reference organisms. Finally, let $\hat h_{g}(R)$ and $\hat h_{g}(S)$ be the (observed) hamming distance WRT gene *g* between the reference organisms (*R*_1_ and *R*_2_) and between the strains (*S*_1_ and *S*_2_), respectively. Similarly, let $\hat h_{w}(R)$ and $\hat h_{w}(S)$ be the hamming distance between the reference organisms WRT the witness gene *w*. Then the probability of observing $\hat h_{g}(S)$ given $\hat h_{g}(R)$, $\hat h_{w}(R)$, $\hat h_{w}(S)$ and *n* assuming CRM hypothesis is:

11 $$ Pr\left(\left|\hat h_{g}(s) - corr^{-1}(d_{g}(S))\right|>\varepsilon\right) \leq 2e^{-2n\varepsilon^{2}},  $$

where, *d*_*g*_(*S*)is the expected distance between the strain species *S*_1_ and *S*_2_ given by

$$d_{g}(S)=\frac{corr\left(\hat h_{g}(R)\right)}{corr\left(\hat h_{w}(R)\right)}{corr\left(\hat h_{w}(S)\right)}$$

##### *Proof*

We first use $\hat h_{g}(R))$ and $\hat h_{w}(R)$ to compute $\rho = \frac {corr(\hat h_{g}(R))}{corr(\hat h_{w}(R))}$ and then by Observation 3 we obtain expected distance between the strains *d*_*g*_(*S*). Finally, by using the inverse correction on *d*_*g*_(*S*) we can use Lemma 2 to bound the probability of the deviation of the observed Hamming distance between the strains, *h*_*g*_(*s*), from the expected one. □

Using Theorem 1 we can find a cutoff value for the difference between the expected and observed Hamming distances for any given confidence level *δ*_*r*_:

##### **Corollary 3**

For a given confidence value *δ*_*r*_, we can refute the null hypothesis, i.e., that gene *g* has evolved vertically, if the difference |*h*_*g*_(*s*)−*corr*^−1^(*d*_*g*_)| satisfies:

12 $$ |h_{g}(s) - corr^{-1}(d_{g})|> \varepsilon(\delta_{r}) = \sqrt{\frac{-\log_{e} \delta_{r}/2}{2n}}  $$

##### *Proof*

Equation (11) gives the probability for a deviation from the expected distance *d*_*g*_. The bigger the deviation, the smaller is its probability. Therefore we can calculate the minimum deviation *ε*(*δ*_*r*_) with probability at most the threshold value *δ*_*r*_, and refute the null hypothesis for any bigger deviation. □

**Fig. 3:**
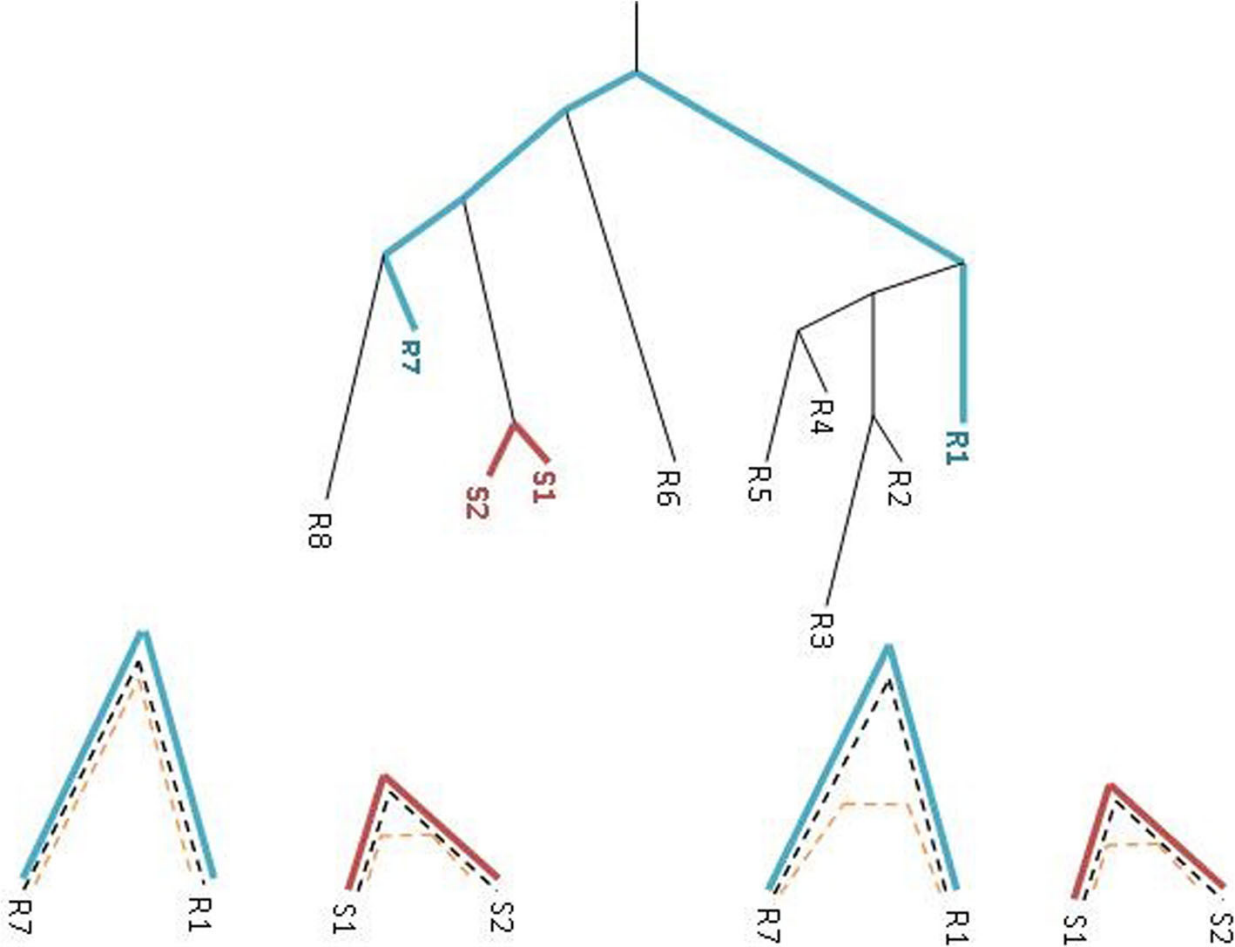
The phylogeny over a group of organisms with branch lengths proportional to distances of gene *g*_*h*_. *g*_*h*_ has undergone HGT between the two strains *S*_1_ and *S*_2_ and hence their distance is very short compared with two reference organisms *R*_1_ and *R*_7_. Bottom: The reference gene (blue, dashed line) must be a gene that accumulates mutations ever since the divergence of both the strains and reference organisms. There are two cases in which the suspicious gene evolves at the reference organism. (*A*) No HGT and then the constant relative conservation is maintained. (*B*) HGT of the SI suspicious gene at the reference organisms and the constant relative conservation is not maintained

Figure 3 illustrates the situation. The following example applies Theorem 1 under the Jukes–Cantor [15] (JC) evolutionary model, on real data from the *E. coli* strains *CFT073* and *MG1655* [1].

##### **Example 1**

To illustrate the use of Theorem *1* we show some real data example.

The evolutionary model with which we work is the Jukes–Cantor *[*15*]* (JC) evolutionary model. The JC model is a reversible, one parameter model, postulating that at any position the rate of substitutions from one state to another, *a*_*i*,*j*_ is the same – *a*. Under this model, the expected number of substitutions – that is, the evolutionary distance *d*_*JC*_ – at a site during *t* time units is *d*_*JC*_=3*at*. To obtain the distance from a given Hamming distance *h*,we apply the distance correction for the JC model:

13 $$ d_{JC}=corr_{JC}(h)=-\frac{3}{4}\log_{e}\left(1-\frac{4}{3}h\right),  $$

for a given normalized Hamming distance *h*. Note that under this correction, $h < \frac 34$ must hold.

Inversely, the expected Hamming distance is:

14 $$ h =corr^{-1}_{JC}(d_{JC})=\frac{3}{4}\left (1-e^{-\frac 43d_{JC}}\right).  $$

Let the two strains *S*_1_ and *S*_2_ be the *E. coli* strains *CFT073* and *MG1655* and the reference organisms, *R*_1_ and *R*_2_, be *Bacteroides.fragilis* and *Wolbachia*. The HGT suspected gene is *engA* and the witness gene is *gmk*. The Hamming distances obtained are: *h*_*g*_(*s*)≈0.0237, *h*_*g*_(*r*)≈0.583, *h*_*w*_(*r*)≈0.541, *h*_*w*_(*s*)≈0.008 and the average genome size is 4743. We get:

- *h*_*g*_(*s*)=0.0237
- *h*_*g*_(*r*)=0.583
- *h*_*w*_(*r*)=0.541
- *h*_*w*_(*s*)=0.008
- *n*=1472.

we get:

$$\begin{aligned} d_{g}(s)=&\frac{corr_{JC}(h_{g}(r))}{corr_{JC}(h_{w}(r))}{corr_{JC}(h_{w}(s))}\\ =&\frac{corr_{JC}(0.583)}{corr_{JC}(0.541)}{corr_{JC}(0.008)}\\ =&\frac{-\frac{3}{4}\log_{e}\left (1-\frac{4}{3}\cdot0.583\right)}{-\frac{3}{4}\log_{e}\left (1-\frac{4}{3}\cdot 0.541\right)} *\frac{3}{4}\log_{e}\left (1-\frac{4}{3}\cdot0.008\right)\approx0.0095. \end{aligned} $$

Hence:

$$\begin{array}{*{20}l} h_{g}(s)-corr_{jc}^{-1}(d_{g}(s))&=0.0237-\left (\frac{3}{4}\left (1-e^{-\frac{4}{3}\cdot0.0095}\right)\right)\\&=0.0237 - 0.0094 \approx 0.0142 \end{array} $$

and the probability to see a difference greater than 0.0142 is

$$Pr(|h_{g}(s) - corr^{-1}(d_{g})|>0.0142) \leq2e^{-2n\cdot0.0142^{2}} \approx 0.85. $$

We can see that for such a difference, the null hypothesis is not rejected and we cannot refute by gene *gmk* that gene *engA* evolved vertically.

Alternatively, if we set *δ*_*r*_=0.05 then by Eq. (*12*) we get a cutoff distances *ε*(0.05)

$$ \varepsilon(0.05) = \sqrt{\frac{-\log_{e} 0.05/2}{2\cdot 1472}} \approx 0.035, $$

which is greater than the value 0.0121 practically obtained.

Theorem 1 gives the tail probability for all events with $h^{\prime }_{g}(s)$, such that $h^{\prime }_{g}(s) \ge h_{g}(s)$. We refute the null hypothesis (that the gene has evolved vertically between the strains) if the probability is below some threshold value *δ*_*r*_.

We conclude this part with the high–level algorithm ***SI–HGT***.


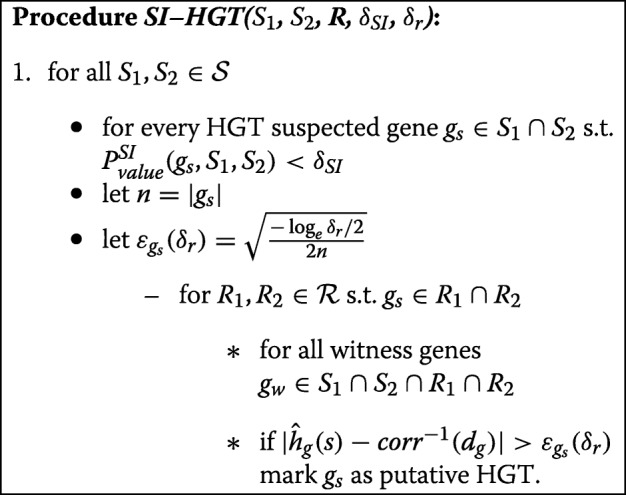


It is important to note here that since we perform many tests for many witness genes and reference organisms, a correction for multiple tests bias should be done. We chose the standard *Bonferroni* correction by multiplying the bound obtained by the number of tests for a given gene.

### Simulation study

We conducted a simulation study to assess the advantage of the new *chernoff* approach over the simple *χ*^2^ approach of [1]. For that we created a simulation process as follow. At first, we simulated a random genome, i.e., a list of genes, which were named based on their order, and we created a random nucleotide sequence for each gene. We also set a mutation rate for each gene, drawn from a normal distribution with given mean and standard deviation. These three parts (gene names, gene sequences, gene mutation rates) constitute a genome object, which will be marked *GA*. Next, we created another three genomes, based on *GA*. Two of them will serve as witness genomes (marked as *GWA* and *GWB*) with identical gene order. Each gene sequence of the witness genomes was created as a copy of the corresponding gene in *GA*, then it had undergone a point mutation performed in accordance with its given mutation rate. The third genome, *GB*, was created at first as a copy of *GA*, then each gene also undergone a point mutation performed in accordance with its given mutation rate. Then *GB* had undergone a genome rearrangement process which was executed as follow. In each round, a gene was randomly chosen as well as neighborhood size. This gene, along with his neighborhood, was swapped with other randomly chosen gene with identical neighborhood size. The neighborhood size was randomly chosen from a normal distribution with a given mean and standard deviation. In addition, each gene in the neighborhoods was undergone a point mutation process accordance with its given mutation rate multiply by some *HGT mutation rate factor*, represents the fact that genes undergone HGT event tend to be more evolutionary distant. When dealing with neighborhoods swapping we refer the genome as a circle. At the end of this process we left with 4 genomes and we can execute our HGT detection algorithm to find the genes undergone rearrangement events in *GB* in relative to *GA*, while *GWA* and *GWB* serve as witness genomes. By this process we could test each approach in terms of false positive (FP, genes which the method identified incorrectly as involved in a rearrangement event).

**Fig. 4:**
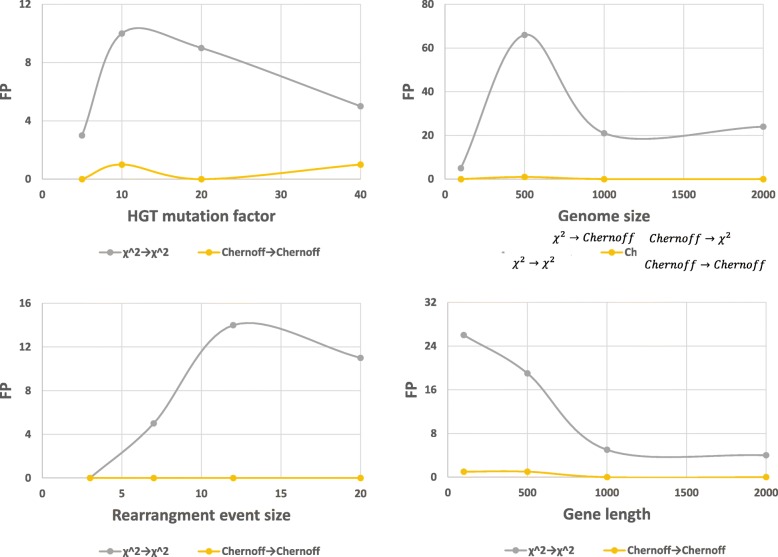
Performance comparison between *χ*^2^ and *Chernoff* approach for SI-based HGT detection under simulated data. Four artificial genomes were created, one served as original genome, one undergone rearrangement events and two served as witness genomes. The two SI-based approaches for HGT detection were executed and were compared in terms of False positive (FP). Number of HGT events- 10. Each point represents an average of 10 repeats. Default parameters: genome size=1000, gene length=1000, HGT mutation factor=10, rearrangement event size=7

We performed this simulation in which the two approaches of HGT detection (*χ*^2^ and *chernoff*) were competed in terms of False positive events and results are shown in Fig. 4. As can be seen, *chernoff* approach presents much better results in terms of FP, i.e., this configuration yield only few genes which was actually not involved in any rearrangement event, especially for short genomes, while the *χ*^2^ approach presents relatively high FP value. This is an expected outcome, in light of the inherited permissive nature of the *χ*^2^ approach.

### Real data study

**Fig. 5:**
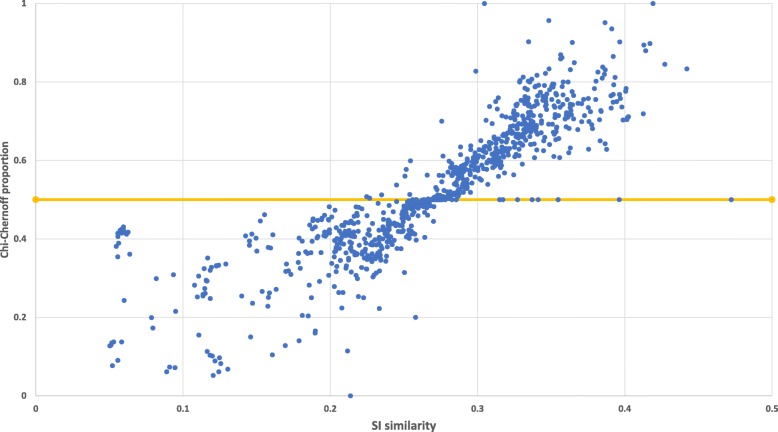
Performance comparison between *χ*^2^ and *Chernoff* approach for SI-based HGT detection under real biological data. Both the *Chernoff*-based approach and the *χ*^2^ approach for HGT detection were applied on real biological data set contains 1229 pair of closely related species from Eggnog repository. Each point represent a pair of species. The X axis is the SI similarity among the pair. The Y axis is the proportions of the number of HGT-genes detected by the *χ*^2^ approach divided by the amount of genes detected by both of the approaches (i.e., high value in the Y axis represents more genes detected by the *χ*^2^ approach then by the *Chernoff* approach. The yellow line represent the boundary between the two cases)

In order to demonstrate the new HGT–detection method based on *Chernoff* bound we applied it on large real data set from EggNog repository [22], and compared the results to our previous approach based on *χ*^2^. The set contains 1229 pairs of bacteria, in which all pairs are of the same taxonomy genus and species (for example, one of the pairs is *Acinetobacter baumannii AB0057* and *Acinetobacter baumannii AB307–0294*). As can be seen in Fig. 5, we found that for closely related species (SI <0.27), the *χ*^2^ approach detects more genes than *Chernoff*–based approach. We assume that the other genes are identified by the *χ*^2^–based approach are mostly false positive, and this finding of HGT–detection in closely related species is consistent with the simulations presented above (Fig. 4, which presents the low false positive cases of the *Chernoff*–based approach as its main advantage). For less related species, *Chernoff*–based approach detects more HGT–genes than the *χ*^2^ approach, and we assume most of the differences are false positive cases, and this might be a results of high neighborhood size which results high calculated threshold for this low synteny similarity presented by these non–related species (see Eq. (6)). We end this section by recommendation of using this new approach for HGT–detection among closely related species.

## Discussion and conclusions

In this work we have provided a probabilistic approach to detect HGTs based on the *synteny index* (SI) and the *constant relative mutability* (CRM) that were defined in [1]. The advantage of the approach portrayed here is the quantification of the statistical signal and using probabilistic bounds to decree significance. The first contribution of this work is assessment of the significance of the SI of a gene. This is essential as distantly related genomes exhibit low SI by default and therefore it is required to distinguish between background noise to signal. The next step is a rigorous probabilistic formulation of the HGT under the CRM property, such that deviations from expected values can be detected and quantified. This requires switching between two spaces– the hamming distance space where bounds on deviations are employed, and the mutation distance space where the CRM property holds. We showed by simulation that the new approach provides greater specificity (i.e., lower false positive rate) over the *χ*^2^ criterion that was provided by [1]. We also demonstrated the specificity improvement in real biological data set. We comment that, as was demonstrated in [1], the advantage of the SI based approach over existing HGT detection techniques, is between closely related taxa where the signal is weak whatsoever. Therefore the improvement in the specificity is imperative. We comment that all steps performed in the algorithm are very fast. One bottleneck in the implementation is the identification of orthologous genes, however the same obstacle stands also in other approaches. Future directions we see in this direction include the establishment of a special repository holding the genes found as HGT putative, similarly to the HGT–DB [10]. Another challenging task is the identification of orthologous genes across many species. This problem stands at the heart of almost any task in comparative genomics. The novelty of our approach is the consideration of gene order among the genomes. While this order can serve as informative for detecting orthology, its lack thereof can allude to exceptional events such as HGT.

## Data Availability

All real data used in this study was taken from EggNOG repository, which is a public online sources, available at: http://eggnog.embl.de/version_3.0/.

## Abbreviations

COG: Cluster of orthologous Groups
CRM: Constant relative mutability
EggNog: Evolutionary genealogy of genes non supervised orthologous groups
HGT: Horizontal gene transfer
SI: Synteny index
UPM: Universal pacemaker
